# LC-MSMS based screening of emerging pollutant degradation by different peroxidases

**DOI:** 10.1186/s12896-019-0574-y

**Published:** 2019-11-28

**Authors:** Khadega A. Almaqdi, Rana Morsi, Bahia Alhayuti, Farah Alharthi, S. Salman Ashraf

**Affiliations:** 1 Department of Chemistry, College of Science, UAE University, Al Ain, UAE; 2 Department of Chemistry, College of Arts and Sciences, Khalifa University, P O Box 127788, Abu Dhabi, UAE

**Keywords:** Emerging pollutants, Soybean peroxidase, Chloroperoxidase, Lactoperoxidase, Manganese peroxidase, Horseradish peroxidase, Redox mediator, Furosemide and trimethoprim

## Abstract

**Background:**

The presence of a wide range of bioactive organic pollutants in wastewater and municipal water sources is raising concerns about their potential effects on humans. Not surprisingly, various approaches are being explored that can efficiently degrade these persistent organic pollutants. Use of peroxidases has recently been recognized as a novel remediation approach that may have potential advantages over conventional degradation techniques. However, testing the abilities of different peroxidases to degrade diverse emerging pollutants is tedious and cumbersome.

**Results:**

In the present study, we present a rapid and robust approach to easily test the degradability of 21 different emerging pollutants by five different peroxidases (soybean peroxidase, chloroperoxidase, lactoperoxidase, manganese peroxidase, and horseradish peroxidase) using an LC-MSMS approach. Furthermore, this approach was also used to examine the role of a redox mediator in these enzymatic degradation assays. Our results show that some of the organic pollutants can be easily degraded by all five of the peroxidases tested, whereas others are only degraded by a specific peroxidase (or when a redox mediator was present) and there are some that are completely resistant to degradation by any of the peroxidases tested (even in the presence of a redox mediator). The degradation of furosemide and trimethoprim by soybean peroxidase and chloroperoxidase, respectively, was investigated in detail by examining the transformation products generated during their degradation. Some of the products generated during enzymatic breakdown of these pollutants have been previously reported by others, however, we report many new transformation products.

**Conclusions:**

LC-MSMS approaches, like the one described here, can be used to rapidly evaluate the potential of different peroxidases (and redox requirements) to be used as bioremediation agents. Our preliminary result shows peroxidases hold tremendous potential for being used in a final wastewater treatment step.

## Background

It is now well-established that “contaminants of emerging concerns” or “emerging pollutants” are increasingly being detected in our water supply. These emerging pollutants comprise an extensive array of diverse compounds and their transformation products, such as nonsteroidal anti-inflammatory drugs (NSAIDs), analgesics, antibiotics, textile dyes, hormones, personal care products and pesticides [1]. A recent study of pesticide contamination due to agriculture activities found significant concentrations of Fluometuron (317.6 μg/L), Chlorpyrifos (0.42 μg/L), and Prometryn (0.48 μg/L) in surface waters of Lake Vistonis Basin, in Greece [2]. Similarly, significant levels of pharmaceuticals (e.g. Lincomycin, Sulfamethoxazole, and Tetracycline) have been detected in U.S. streams as early as 1999 [3]. Not surprisingly, these compounds are suspected to cause a wide array of adverse ecological or human health effects and have become the focus of various government as well as academic research groups [4]. For example, the presence of perfluorinated compounds in the serum has been correlated with breast cancer risk in Greenlandic Inuit women [5]. Additionally, it has been reported that pollutants such as perfluorooctanoate and perfluorooctane sulfonate may be linked to decreased human reproductive abilities [6]. Scientific literature is full of reports of various physical and chemical approaches can that be employed for the removal of these emerging pollutants [7–10]. However, more research is still needed to develop more efficient, economical, and ‘environmental-friendly’ and ‘greener’ remediation approaches.

During the past few years, the role of oxidoreductive enzymes in ‘green processes’ has become more established and not surprisingly various enzyme systems have been employed for the efficient degradation of diverse organic pollutants [11, 12]. Amongst the various advantages offered by enzymatic degradation approach, the most important ones are the mild and less toxic reagents and conditions that are normally employed in their use as well as their ability to degrade a wide range of substrates. The main potential disadvantage with the use of enzymes is their relatively high cost, however, this can be ameliorated using recombinant DNA technology to mass-produce cheaper enzymes. Literature survey shows that various types of pollutants have been degraded by two different classes of enzymes such as laccases and peroxidases such as Soybean Peroxidase (SBP), Manganese Peroxidase (MnP), Lignin Peroxidase (LiP) and Horseradish Peroxidase (HRP) [13–15]. Additionally, peroxidases from other plant sources such as cauliflower, white radish, and turnip, have been used for the degradation of various organic compounds [16–19]. Besides this, peroxidases from bamboo shoots and lemon peel have also been used for degrading dyes [20, 21]. The addition of redox mediator (RM) to the system has shown to enhance the degradation process to produce less toxic substances [22–25]. Despite the relatively large number of reports showing the application of peroxidases for remediation purposes, only a very few studies have carried out detailed and systemic studies comparing the efficiencies of different oxidoreductases (e.g. peroxidases and laccases) towards degrading a wide range of emerging pollutants. This shortage of systematic studies further highlights the cumbersome and tedious nature of these ‘peroxidase-degradability screening’ studies.

The current work describes a sensitive and robust approach using LC-MSMS that was developed to simultaneously quantify a large number of emerging pollutants and to easily examine their degradability by different peroxidases. This approach was also used to examine the effect of redox mediators for efficient peroxidase-mediated degradation of emerging pollutants. Additionally, we report on the transformation products generated during the enzymatic breakdown of furosemide (with SBP) and trimethoprim (with CPO) in the presence of redox mediator HOBT. Interestingly, many of the intermediates observed have not been previously reported for the degradation of these emerging pollutants by other remediation methods.

## Results

### Development of a sensitive LC-MSMS based method for the quantification of 21 emerging pollutants

**Fig. 1:**
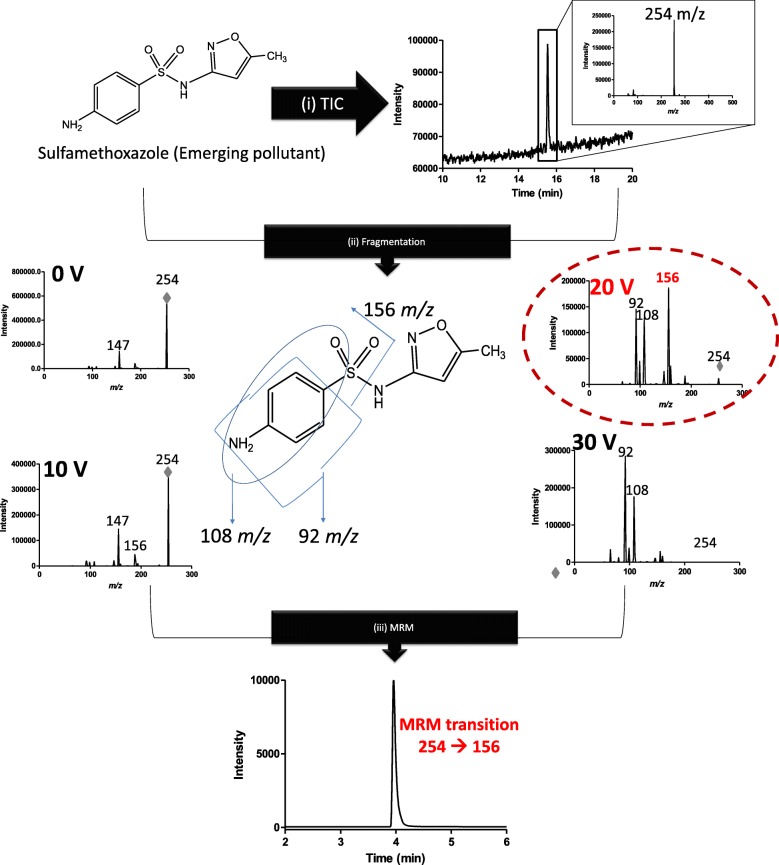
Schematic diagram of the MRM (LC-MSMS) method development for Sulfamethoxazole

HPLC and LC-MS-based methods are widely reported for the detection and quantification of various individual organic compounds, including emerging pollutants. However, since we wanted to simultaneously study the degradation of a large number of different emerging pollutants (24 of them), we first developed a sensitive, robust, and easy LC-MSMS method, using the Multiple Reaction Monitoring (MRM) approach. The MRM method uses tandem mass spectrometers to specifically monitor the “precursor to product transition” generated when a specific emerging pollutant (precursor ion) is fragmented into a specific product ion. Since the detection is based on a specific “precursor ➔ product transition” which is unique to a specific compound, it allows for simultaneous detection of a large number of compounds without having them completely resolved in the liquid chromatography part of the LC-MSMS method [26]. Figure 1 schematically shows the steps that are taken in developing these MRM-based assays (for sulfamethoxazole, for example) – starting with confirming the parental mass of sulfamethoxazole (253 Da) in the LC-MS (when run in “Total Ion Chromatogram” (TIC) mode. This precursor (parent) ion (M + H)^+^ species (254 m/z) is then fragmented in the LC-MS by increasingly higher collision energy values (0 V, 10 V, 20 V, and 30 V were used for sulfamethoxazole). When a significantly high and strong signal for a specific product ion is observed (e.g. 156 m/z), that specific collision energy (e.g. 20 V) and the precursor ➔ product transition (254➔ 156) are then used for the MRM method. There are numerous examples of the use of such MRM-based analyses for organic compounds in various matrices [26, 27].

**Fig. 2:**
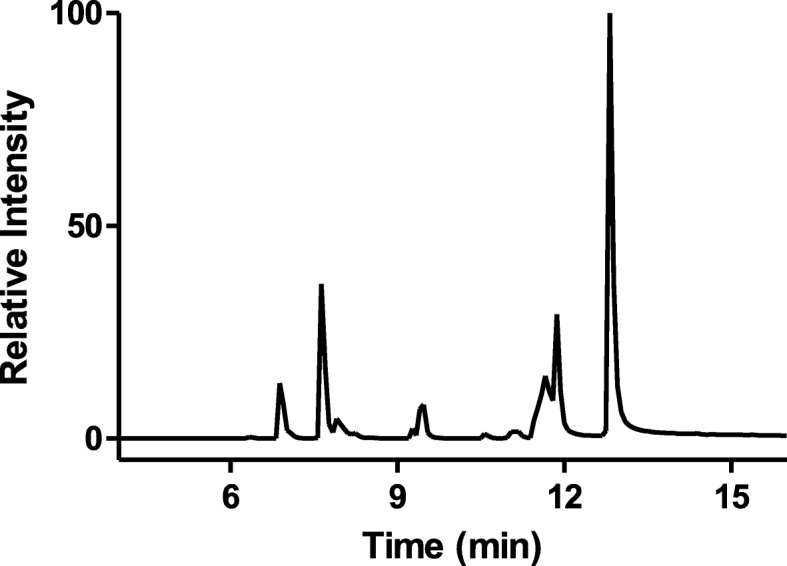
Total MRM scan of a mixture of 21 emerging pollutants (EPs). [EPs] = 2 ppm, [H_2_O_2_] = 0.1 mM, [HOBT] = 0.1 mM, pH = 4

**Fig. 3:**
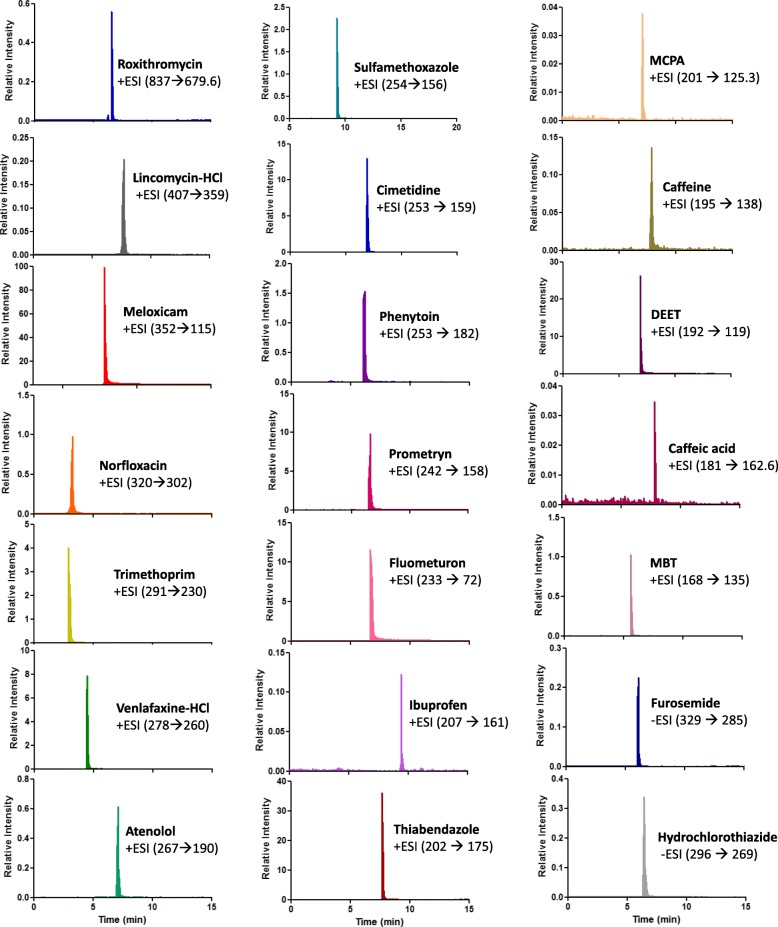
Individual extracted MRM scans for the 21 emerging pollutants (EPs). [EPs] = 2 ppm, [H_2_O_2_] = 0.1 mM, [HOBT] = 0.1 mM, pH = 4

Table 1 shows the categories and structures of these different emerging pollutants, as well as their MRM and mass-spectrometry parameters. Also shown in Table 1 are the retention times on a C_18_ column for these compounds (explained in more detailed in the Methods section). Figure 2 shows the typical chromatogram obtained when a mixture of these 21 emerging pollutants were analyzed using the developed method. The extracted individual chromatograms of these emerging pollutants (concentration 2 ppm) are shown in Fig. 3, which shows the specificity and sensitivity of the analytical assay.

**Table 1:** Names and chemical structures of the 21 emerging pollutants used in this study and their LC-MSMS parameters

	Category	Emerging pollutant	Structure	Retention Time (min)	MRM Transition (m/z)	Dwell time (ms)	Frag-mentor Voltage (V)	Collision Energy (V)	Polarity
1	Antibiotic	Roxithromycin		11.6	837 ➔ 680	200	135	20	Positive
2		Lincomycin-HCl		7.6	407 ➔ 359	200	135	20	Positive
3		Norfloxacin		8.2	320 ➔ 302	200	135	20	Positive
4		Trimethoprim		7.9	291 ➔ 230	200	135	20	Positive
5		Sulfamethoxazole		9.3	254 ➔ 156	200	135	20	Positive
6	Antidepressant	Venlafaxine-HCl		9.4	278 ➔ 260	200	135	10	Positive
7	Antioxidant	Caffeic acid		7.8	181 ➔ 163	200	135	20	Positive
8	Anti-seizure drug	Phenytoin		11.1	253 ➔ 182	200	135	10	Positive
9	Diuretic drug	Hydrochloro-thiazide		6.4	296 ➔ 269	70	140	20	Negative
10		Furosemide		11	329 ➔ 285	70	140	15	Negative
11	Beta-blocker (high blood pressure drug)	Atenolol		7.1	267 ➔ 190	200	135	20	Positive
12	Fungicide	Thiabendazole		7.6	202 ➔175	200	135	30	Positive
13	Herbicide	Prometryn		11.6	242 ➔ 158	200	135	30	Positive
14		MCPA		12	201 ➔ 125	200	47	13	Positive
15		Fluometuron		11.7	233 ➔ 72	200	135	30	Positive
16	Histamine H_2_ receptor antagonist	Cimetidine		6.9	253 ➔ 159	200	135	10	Positive
17	Insect repellent	DEET		11.9	192 ➔ 119	200	135	30	Positive
18	Nonsteroidal anti-inflammatory drug (NSAID)	Meloxicam		12.8	352 ➔ 115	200	135	6	Positive
19		Ibuprofen		14.4	207 ➔ 161	200	135	20	Positive
20	Stimulant	Caffeine		7.8	195 ➔ 138	200	135	30	Positive
21	Vulcanization agent (for rubber)	2-Mercapto Benzothiazole (MBT)		10.6	168 ➔ 135	200	135	30	Positive

### Degradation of emerging pollutants by five different peroxidases

**Fig. 4:**
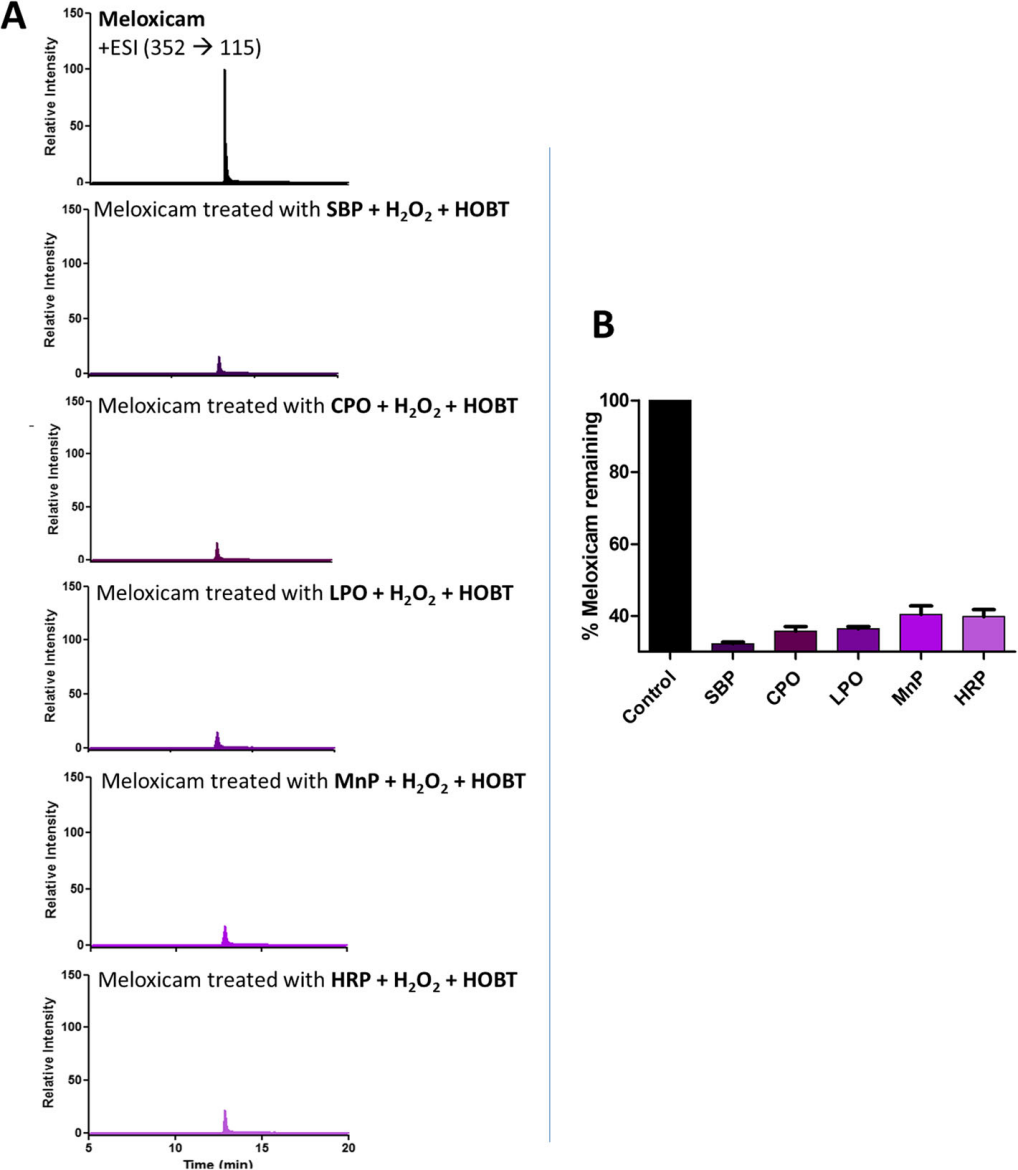
**a** MRM scans of Meloxicam treated with SBP, CPO, LPO, MnP and HRP enzymes. **b** % of Meloxicam remaining after treatment with SBP, CPO, LPO, MnP and HRP enzymes. [Meloxicam] = 2 ppm, [Enzyme] = 0.36 μM, [H_2_O_2_] = 0.1 mM added 3 times of 10 min interval, [HOBT] = 0.1 mM, pH = 2 for CPO, pH = 4 for SBP, pH = 5 for MnP and pH = 6 for LPO and HRP

**Fig. 5:**
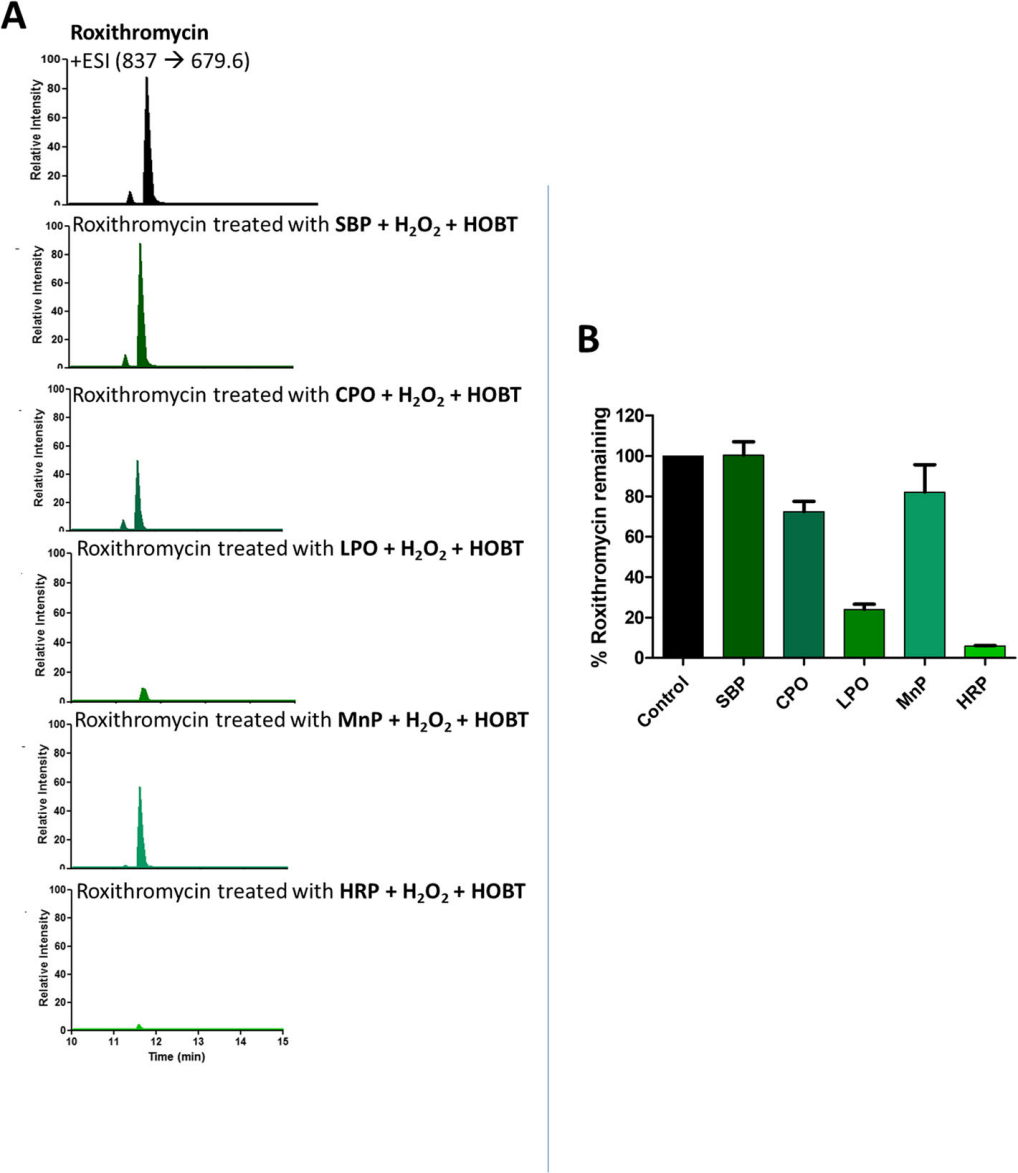
**a** MRM scans of Roxithromycin treated with SBP, CPO, LPO, MnP and HRP enzymes. **b** % of Roxithromycin remaining after treatment with SBP, CPO, LPO, MnP and HRP enzymes. [Roxithromycin] = 2 ppm, [Enzyme] = 0.36 μM, [H_2_O_2_] = 0.1 mM added 3 times of 10 min interval, [HOBT] = 0.1 mM, pH = 2 for CPO, pH = 4 for SBP, pH = 5 for MnP and pH = 6 for LPO and HRP

It has been previously reported that different peroxidases may have different remediation efficiencies for different organic pollutants [28, 29]. Therefore, mixtures of 21 emerging pollutants were separately treated with Soybean Peroxidase (SBP), Chloroperoxidase (CPO), Lactoperoxidase (LPO), Manganese Peroxidase (MnP), or Horseradish Peroxidase (HRP), as described in more detail in the Methods section. Figure 4a and b show the residual amounts of Meloxicam, a nonsteroidal anti-inflammatory drug, that has been found in many water bodies [30], upon treatment by the above five named peroxidases. As can be seen from Fig. 4b, all of the peroxidases tested were able to degrade Meloxicam nicely, with SBP being slightly more efficient than the others. This is consistent with our previous studies showing that SBP could efficiently degrade related thiazole compounds [28]. However, it seems that not all organic pollutants could be equally degraded by the peroxidases tested. For example, Fig. 5a and b show the percentage of Roxithromycin remaining after treatment with the peroxidases. As can be seen, SBP was not able to degrade this compound and CPO and MnP were also not efficient in degrading it, showing only around 25% degradation. However, HRP could easily degrade it and showed almost 95% degradation (in 30 min), as could LPO as well, showing about 80% degradation. These and other differences in the efficacies of different peroxidases to degrade specific emerging pollutants are summarized in Table 2. As can be seen from the table, 8 of the 21 emerging pollutants tested could be degraded very efficiently (> 75% in 30 min) by at least one of the peroxidases, with two more (Thiabendazole and Meloxicam) showing fairly good enzymatic degradation (> 50%).

**Table 2:** A summary of the % of EPs remaining after treatment with five different enzymes SPO, CPO, LPO, MnP and HRP with and without HOBT. Key: %EP remaining 0–25% (+++), 25–50% (++), 50–75% (+), More than 75% (−)

NO.	Category	Emerging pollutant	SBP Only	SBP + HOBT	CPO Only	CPO + HOBT	LPO Only	LPO + HOBT	MnP Only	MnP + HOBT	HRP Only	HRP + HOBT
1	Antibiotic	Roxithromycin	-	-	-	+	-	+++	+	-	+	+++
2	Antibiotic	Lincomycin-hydrochloride	-	-	-	-	-	++	-	-	-	+++
3	Antibiotic	Norfloxacin	-	-	-	-	-	+	-	-	-	+
4	Antibiotic	Trimethoprim	-	-	-	++	-	-	-	-	-	-
5	Antibiotic	Sulfamethoxazole	+	+++	-	-	+	+++	-	-	-	+++
6	Antidepressant	Venlafaxine-hydrochloride	-	-	-	-	-	-	-	-	-	+
7	Antioxidant	Caffeic acid	+++	+++	+	+	+++	+++	+++	+++	+++	+++
8	Anti-seizure drug	Phenytoin	-	-	-	-	-	-	-	-	-	-
9	Diuretic drug	Hydrochlorothiazide	-	-	-	-	-	+	-	-	-	+++
10	Drug for treating fluid build-up due to heart failure, liver scarring, or kidney disease	Furosemide	++	+++	++	+++	-	+++	-	-	-	+++
11	Drug for treating high blood pressure	Atenolol	-	-	-	-	-	-	-	-	-	-
12	Fungicide	Thiabendazole	++	++	++	-	++	++	++	-	++	++
13	Herbicide	Prometryn	-	-	-	-	-	-	-	-	-	-
14	Herbicide	MCPA	-	-	-	-	-	-	-	-	-	-
15	Herbicide	Fluometuron	-	-	-	-	-	-	-	-	-	-
16	Histamine H_2_ receptor antagonist	Cimetidine	++	++	++	-	++	-	++	-	++	+++
17	Insect repellents	DEET	-	-	-	-	-	-	-	-	-	-
18	Nonsteroidal anti-inflammatory drug (NSAID)	Meloxicam	++	++	+	++	++	++	+	++	++	++
19	Nonsteroidal anti-inflammatory drug (NSAID)	Ibuprofen	-	-	-	-	-	-	-	-	-	-
20	Stimulant	Caffeine	-	-	-	-	-	-	-	-	-	-
21	Sulfur vulcanization of rubber	Mercaptobenzothiazole (MBT)	+++	+++	++	++	+++	+++	+++	+++	+++	+++

### Role of redox mediators for efficient degradation of emerging pollutants by peroxidases

**Fig. 6:**
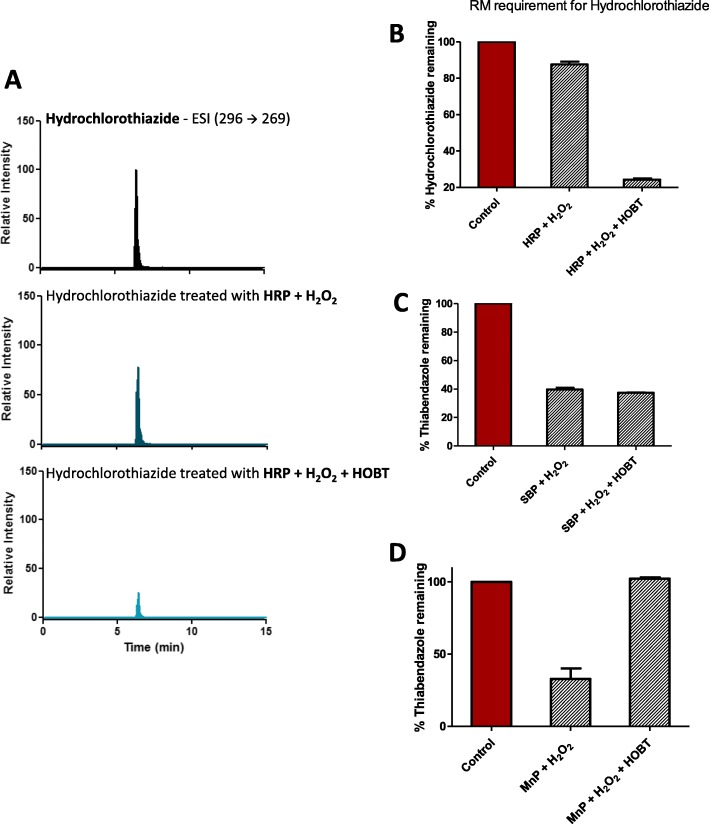
Effect of redox mediator on peroxidase-mediated pollutant degradation. **a** MRM scans of Hydrochlorothiazide treated with HRP enzyme with and without HOBT. **b** % of Hydrochlorothiazide remaining after treatment with HRP enzyme with and without HOBT. [Hydrochlorothiazide] = 2 ppm, [HRP] = 0.36 μM, [H_2_O_2_] = 0.1 mM added 3 times of 10 min interval, [HOBT] = 0.1 mM, pH = 6. **c** % of Thiabendazole remaining after treatment with SBP enzyme with and without HOBT. **d** % of Thiabendazole remaining after treatment with MnP enzyme with and without HOBT. [Thiabendazole] = 2 ppm, [H_2_O_2_] = 0.1 mM added 3 times of 10 min interval, [HOBT] = 0.1 mM, pH = 4 with SBP enzyme and pH = 5 with MnP enzyme, [enzyme] = 0.36 μM

In the present study, we also evaluated the role of a redox mediator for the efficient degradation of our chosen 21 emerging pollutants by the five peroxidases. As can be seen in Fig. 6a and b, Hydrochlorothiazide showed only marginal degradation by HRP + H_2_O_2_, resulting in about 10% degradation in 30 min. However, the presence of 0.1 mM HOBT increased the degradation rate dramatically (to about 75% degradation in 30 min). Similar results were observed with SBP-mediated degradation of Sulfamethoxazole, which also required HOBT for efficient degradation [31]. However, such a dramatic enhancement of organic pollutant degradation by the redox mediator, HOBT, was not universal. For example, SBP + H_2_O_2_ could degrade about 55% of Thiabendazole in 30 min, however, the addition of HOBT had no beneficial effect on the degradation of this fungicide (Fig. 6c and Additional file 1: Figure 6CS). Interestingly, the inclusion of HOBT could also hinder the peroxidase-mediated degradation of these compounds. This is dramatically seen for Manganese Peroxidase mediated degradation of Thiabendazole, where the presence of HOBT completely inhibited its degradation (Fig. 6d and Additional file 1: Figure 6DS). In fact, these inhibitory effects of redox mediators are not unexpected as these redox mediators can bind to and react with peroxidases with high affinity and thereby compete with organic pollutants for binding. In fact, our lab has been previously reported that CPO-mediated chlorination of ThT showed a significant decrease in the presence of HOBT [28]. Nevertheless, 8 of the compounds tested appeared to be completely recalcitrant to degradation by any of the five peroxidases tested, even in the presence of the redox mediator, HOBT (Table 2).

### SBP-mediated degradation of emerging pollutants in a real wastewater sample

We also wanted to use our newly developed approach to carry out a preliminary and exploratory study to screen a real wastewater sample for the presence of emerging pollutants and to test their degradation by one of the peroxidases. LC-MSMS analysis of a sample of pretreatment wastewater feed from the local municipal wastewater treatment plant showed the presence of two different emerging pollutants, namely, meloxicam and DEET. The presence of DEET in wastewater feed has been previously reported in China by Sui et al. [32], whereas meloxicam and many other emerging pollutant have been detected in surface water as well as in wastewater treatment plant in Serbia [33].

**Fig. 7:**
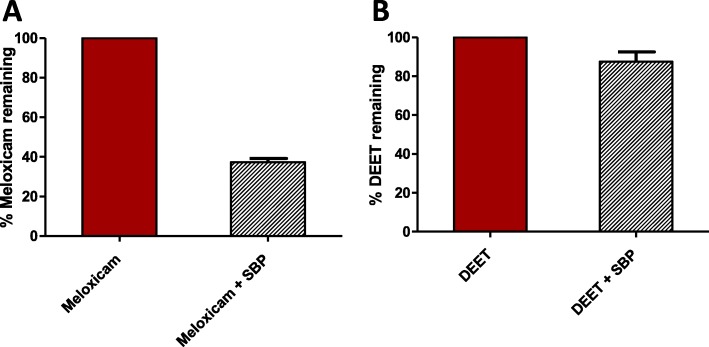
Degradation of pollutants spiked in real wastewater. **a** % of meloxicam remaining after treatment with SBP enzyme. **b** % of DEET remaining after treatment with SBP enzyme. [SBP] = 1.56 μM, [H_2_O_2_] = 0.1 mM, [HOBT] = 50 μM, pH = 4

Treatment of the local municipal wastewater sample treatment produced results very similar to those obtained with neat solutions (Table 2). As can be seen in Fig. 7, SBP was able to degrade about 50% of the meloxicam in the wastewater sample in 30 min, whereas no significant degradation was observed for the DEET present in the sample (Additional file 1: Figure 7AS and figure 7BS). Although no optimization steps were carried out in this exploratory study with real wastewater, these preliminary experiments confirmed the results obtained with neat pollutants. Furthermore, the results also support the potential applicability of peroxidase enzymes to degrade some organic pollutants in complex matrixes, such as wastewater samples.

### Peroxidase-mediated degradation of furosemide and trimethoprim and transformation product identification

**Fig. 8:**
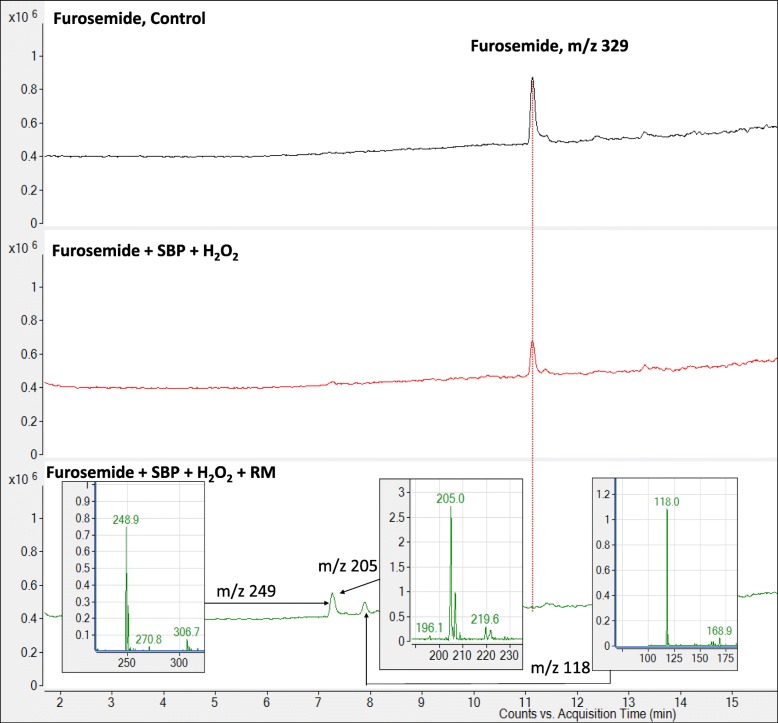
LC-MS total ion chromatograms of furosemide treated with SBP, SBP + H_2_O_2_, and SBP + H_2_O_2_ + HOBT

**Fig. 9:**
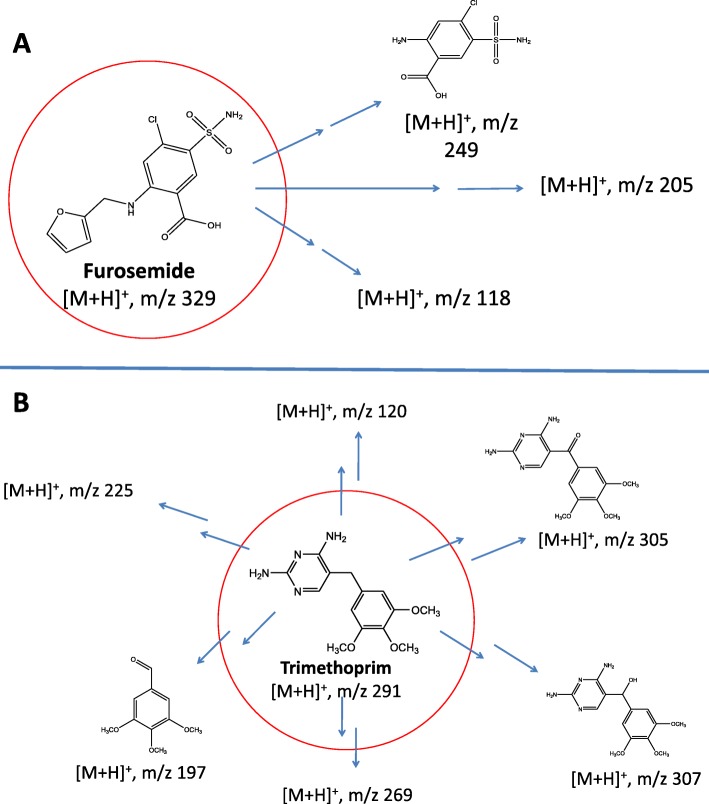
**a** Proposed degradation pathways of furosemide by SBP + H_2_O_2_ + HOBT. **b** Proposed degradation pathways of trimethoprim by CPO + H_2_O_2_ + HOBT

Although the use of mixture of emerging pollutants allowed for rapid and simultaneous screening of peroxidase-mediated degradation of various compounds, no specific details could be obtained about the nature of the degradation products generated. Therefore, we decided to carry out detailed studies with two of the emerging pollutants (furosemide and trimethoprim) to examine the transformation products generated upon their degradation. Addition of SBP + H_2_O_2_ to neat furosemide could degrade about 40% of the diuretic drug, interestingly this could be drastically improved to 100% degradation when the redox mediator HOBT was added to the reaction mixture. This is more clearly seen in the LC-MS total ion chromatogram (Fig. 8), where the furosemide peak decreases upon the addition of SBP + H_2_O_2_ and is completely gone when HOBT was added. These results are consistent with what was observed for furosemide in a mixture with 20 other compounds (Table 2). Furthermore, it can be seen in the chromatogram for the “furosemide + SBP + H_2_O_2_ + HOBT” sample, that decrease of the furosemide peak was accompanied by the appearance of several minor peaks, suggesting the generation of degradation intermediates. The insets in Fig. 8 show the new transformation products to have the m/z ratios of 249, 205 and 118. Fig. 9a shows a summary of the degradation scheme of furosemide by SBP, with the proposed structure of the 249 m/z intermediate. A number of research groups have shown that furosemide can be degraded into smaller breakdown products by various other remediation approaches, including photodegradation [34, 35], electro-Fenton and bioremediation [36]. Table 3 summarizes of these degradation studies, showing the various furosemide breakdown products that have been previously reported. It is interesting to note the 249 m/z species that we observed in our degradation experiments has also been reported earlier by [34, 35].

**Table 3:** Summary of transformation products generated upon the degradation of furosemide and trimethoprim, using AOPs (previous studies) or enzymatic treatment (present study)

	This study	Previous studies	Reference
Furosemide transformation products			
Enzymatic (Soybean Peroxidase)	m/z 118, 205, **249**		Present study
Photodegradation		m/z **249**, 311, 352 and 555	[34]
Photodegradation		m/z 231, 251, **249**, 295, 311 and 329	[35]
Electro-Fenton + Bioconversion		m/z 251,329 and 345	[36]
Trimethoprim transformation products			
Enzymatic (Horseradish Peroxidase)	m/z 120, **197**, 225, 269, **305**, **307**		Present study
Solar-photodegradation		m/z 139, 141, 155, **197**, 213, **305**, **307** and 337	[37]
UVA/LED/TiO2 photocatalysis		m/z 139, 141, **305**, **307**, 323, 325 and 341	[38]
Fenton		m/z 143, 195, 279, 324 and 325	[39]

A similar study was carried out for the degradation of the antibiotic trimethoprim by Chloroperoxidase - the only peroxidase (of the five tested) that could degrade this compound. As shown in Table 2, degradation of trimethoprim by CPO needed the presence of the redox mediator HOBT (data not shown). LC-MSMS analysis of the degradation reaction showed that 6 different transformation products could be detected (Additional file 1: Figure 9BS). Table 3 and Fig. 9b show a summary of the results of the enzymatic degradation of trimethoprim by CPO. Comparison of the transformation products generated during CPO-mediated degradation of trimethoprim with previously published degradation studies of this pollutant [37–39] showed that 3 of the previously published trimethoprim breakdown products were also detected in our study with Chloroperoxidase.

## Discussion

As has been previously reported, Soybean Peroxidase (SBP) and Chloroperoxidase (CPO) show different efficiency and specificity in degrading a given thiazole pollutant [28, 29]. We wanted to extend that observation to three additional peroxidases and examine a total of 21 different emerging pollutants. Our results clearly showed remarkable differences in the specificity and degradability of a range of organic pollutants by different peroxidases. It is tempting to speculate that such differences are primarily related to binding efficiencies of these compounds in the active sites of the peroxidases. Perhaps, future detailed structural studies of the pollutant-peroxidase complexes will shed some light on these interesting observations.

It is also well established that small, diffusible redox mediators can significantly enhance the oxidative degradation of organic pollutants by peroxidases. For example, it has been previously shown that Thioflavin T, a model thiazole pollutant, could only be degraded by SBP in the presence of the redox mediator, 1-hydroxybenzotriazole (HOBT) [28]. Interestingly, as reported earlier [28], addition of HOBT could also inhibit the enzymatic remediation of a few compounds, thus indicating that for some pollutants, HOBT can act as a competitor inhibitor.

The surprising observation that some of the emerging pollutants (8 of the 21 tested) could not be easily degraded by enzyme-mediated oxidative reactions (Table 2), suggest that these enzymatically recalcitrant compounds may require other remediation approaches, such as advanced oxidative processes (AOPs). In fact, we have recently shown that both UV-H_2_O_2_ photolytic AOP and peroxidase-mediated approaches could both be used for the efficient degradation of Sulfamethoxazole, however, they appear to be based on different mechanistic degradation pathways [31]. Perhaps, the enzyme-based degradation approaches could be used in tandem with other AOP-based treatments in wastewater treatment plants to ensure complete and efficient degradation of diverse kinds of emerging pollutants. Our additional experiments with two selected pollutants showed that enzymatically degraded pollutants generated some of the same intermediates that have been previously reported by other remediation methods. However, in the present study, we reported two new transformation products from furosemide that have not been reported previously, namely the m/z 205 and m/z 118. Similarly, we also present three new trimethoprim transformation products (obtained during CPO-mediated degradation) that had not been reported earlier (m/z 120, 225, and 269).

## Conclusions

In summary, we present here a rapid, robust and easy approach to test several oxidative enzymes (peroxidases, laccases, etc.) for their abilities to be used as remediating agents in degrading a large number of emerging pollutants. Our experiments with five different peroxidases show that different enzymes show varying abilities to degrade specific organic compounds. The described LC-MSMS approach was also be used to examine the requirement for redox mediators (HOBT) for peroxidase-mediated degradation of organic pollutants. Additionally, we report that surprisingly, 8 of the 21 emerging pollutants appear to be completely recalcitrant to oxidative degradation by the five peroxidases tested. Finally, we show that degradation studies of two different pollutants (neat and individually) by the peroxidases showed similar results as in a mixture. We also used LC-MSMS to identify some of the furosemide and trimethoprim products generated during their degradation studies using SBP and CPO, respectively.

## Methods

### Reagents and enzymes

All emerging pollutants were obtained from Sigma-Aldrich. Solvents used in LC-MS like LC-MS grade water, acetonitrile, and formic acid as well as Hydrogen peroxide was purchased from Sigma-Aldrich. Universal buffers were used in all experiments (0.2 M potassium phosphate (K_2_HPO4) and 0.1 M citrate acid). The specific enzymes activity for SBP, CPO, LPO MnP and HRP were 2700 IU/mg (1 mg/mL, 26 μM), 1296 IU/mg (17 mg/mL, 405 μM), (10 mg/mL, 26 μM), 200 IU/g (1 mg/mL, 26 μM) and 279 IU/mg (1 mg/mL, 26 μM) respectively. The enzymes (SBP, CPO, and LPO) were purchased from Bio-Research Products (North Liberty, USA). The enzymes (MnP and HRP) they were purchased from Sigma-Aldrich.

### LC-MSMS method development

After treating the 21 emerging pollutants with the five different enzymes SBP, CPO, LPO, MnP, and HRP the samples were analyzed using LCMS. The samples were filtered before injecting them in the LCMS using a 0.45 μm cellulose acetate syringe filter. The column used for analysis was C_18_ column (ZORBAX Eclipse Plus). The column had the following characteristics 1.8 μm particle size, 2.1 mm inner diameter and its length was 50 mm. For the C18 column, its temperature was maintained at 35 °C. The Mass Spectrometry used was 6420 Triple Quad detector (Agilent Technologies). The flow rate for the mobile phase in the column was 0.4 mL/min. The method developed used two mobile phases: 1. (mobile phase A) which was LCMS grade water with 0.1% LCMS grade formic acid 2. (mobile phase B) which was 100% LCMS grade acetonitrile. The method in the multiple reaction monitoring (MRM) analysis was set as follows: 2.5 min of 100% A and 0% B, followed by a 0–80% gradient of B from 2.5–15 min, then at 15.1 min A was 10% and B was 90% for 3 min and finally 2 min of 95% A and 5% B. Positive and negative polarity mode was used for the LC-MSMS experiments depending on the EPs. For the MSMS system, nitrogen gas was used in fragmentation, the capillary voltage, the gas flow, the gas temperature, and the nebulizer pressure were kept at 4000 V, 8 L/min, 3000C and 45 psi, respectively.

### Emerging pollutant degradation and analysis

Twenty-one different emerging pollutants were treated with five different enzymes in the presence and absence of redox mediator. The degradation experiments were done as follows: SBP, CPO, LPO, MnP and HRP enzymes (0.36 μM) were added to 21 EPs (2 ppm) + H_2_O_2_ (0.1 mM). The experiments were carried out in universal buffer, pH = 2 for CPO, pH = 4 for SBP, pH = 5 for MnP and pH = 6 for LPO and HRP. With the redox mediator experiment, 1-hydroxybenzotriazole (0.1 mM) was added to the reaction mixture.

For degradation of emerging pollutants in wastewater, 3 mL of wastewater sample was adjusted to pH 4 using a buffer and then treated with SBP, H_2_O_2_ and HOBT for 30 min ([SBP] = 1.56 μM, [H_2_O_2_] = 0.112 mM, [HOBT] = 0.05 mM, pH = 4). The sample was then filtered and analyzed on LC-MSMS as described above.

Emerging pollutant degradation was represented as “% remaining” and calculated using the “Area Under the Curve” (AUC) of the peaks in the LC-MS-MS spectra (MRM mode), as follows:

$$ \%\mathrm{compound}\ \mathrm{remaining}=\left({\mathrm{AUC}}_{\mathrm{i}}/{\mathrm{AUC}}_{\mathrm{f}}\right)\mathrm{x}100 $$

Where AUC_i_ = AUC of the compound peak in the presence of peroxidase, HOBT and buffer and.

AUC_f_ = AUC of the compound peak in the presence of peroxidase, buffer, HOBT, and H_2_O_2_.

## Additional file

**Additional file 1.** **Figure 6C S**: Effect of redox mediator on peroxidase mediated pollutant degradation. Thiabendazole remaining after treatment with SBP enzyme with and without HOBT. [Thiabendazole ] = 2 ppm, [H 2 O 2 ] = 0.1 mM added 3 times of 10 min interval, [HOBT] = 0.1 mM , pH = 4 with SBP enzyme and pH = 5 with MnP enzyme, [enzyme] = 0.36μM. **Figure 6D S**: Effect of redox mediator on peroxidase-mediated pollutant degradation. Thiabendazole remaining after treatment with MnP enzyme with and without HOBT. [Thiabendazole] = 2 ppm, [H2O2] = 0.1 mM added 3 times of 10 min interval, [HOBT] = 0.1 mM, pH = 4 with SBP enzyme and pH = 5 with MnP enzyme, [enzyme] = 0.36μM. **Figure 7A S**: Degradation of pollutants spiked in real wastewater. Meloxicam remaining after treatment with SBP enzyme. [SBP] = 1.56μM, [H2O2] = 0.1 mM, [HOBT] = 50 μM, pH = 4. **Figure 7B S**: Degradation of pollutants spiked in real wastewater. DEET remaining after treatment with SBP enzyme. [SBP] = 1.56μM, [H2O2] = 0.1 mM, [HOBT] = 50 μM, pH = 4. **Figure 9B S**: Degradation of Trimethoprim by CPO + H2O2 + HOBT.

## Data Availability

All data generated or analyzed during this study are included in this published article [and its supplementary information files].

## Abbreviations

AOP: Advanced Oxidation Process
CPO: Chloroperoxidase
H_2_O_2_: Hydrogen Peroxide
HOBT: 1-hydroxybenzotriazole
HRP: Horseradish peroxidase
LC-MSMS: Liquid Chromatography-tandem Mass Spectrometry
LiP: Lignin peroxidase
LPO: Lactoperoxidase
MnP: Manganese peroxidase
MRM: Multiple Reaction Monitoring
NSAIDs: Nonsteroidal anti-inflammatory drugs
RM: Redox mediator
SBP: Soybean peroxidase
