# Supplementary material for: LC-MSMS based screening of emerging pollutant degradation by different peroxidases
Source: BMC Biotechnol. 2019 Nov 28;19:83. doi: 10.1186/s12896-019-0574-y (PMC6883607; doi:10.1186/s12896-019-0574-y)
Supplement: Supplementary file 1 — Additional file 1. Figure 6C S: Effect of redox mediator on peroxidase mediated pollutant degradation. Thiabendazole remaining after treatment with SBP enzyme with and without HOBT. [Thiabendazole ] = 2 ppm, [H 2 O 2 ] = 0.1 mM added 3 times of 10 min interval, [HOBT] = 0.1 mM , pH = 4 with SBP enzyme and pH = 5 with MnP enzyme, [enzyme] = 0.36μM. Figure 6D S: Effect of redox mediator on peroxidase-mediated pollutant degradation. Thiabendazole remaining after treatment with MnP enzyme with and without HOBT. [Thiabendazole] = 2 ppm, [H2O2] = 0.1 mM added 3 times of 10 min interval, [HOBT] = 0.1 mM, pH = 4 with SBP enzyme and pH = 5 with MnP enzyme, [enzyme] = 0.36μM. Figure 7A S: Degradation of pollutants spiked in real wastewater. Meloxicam remaining after treatment with SBP enzyme. [SBP] = 1.56μM, [H2O2] = 0.1 mM, [HOBT] = 50 μM, pH = 4. Figure 7B S: Degradation of pollutants spiked in real wastewater. DEET remaining after treatment with SBP enzyme. [SBP] = 1.56μM, [H2O2] = 0.1 mM, [HOBT] = 50 μM, pH = 4. Figure 9B S: Degradation of Trimethoprim by CPO + H2O2 + HOBT. [file 12896_2019_574_MOESM1_ESM.pdf]

Fig 6C S

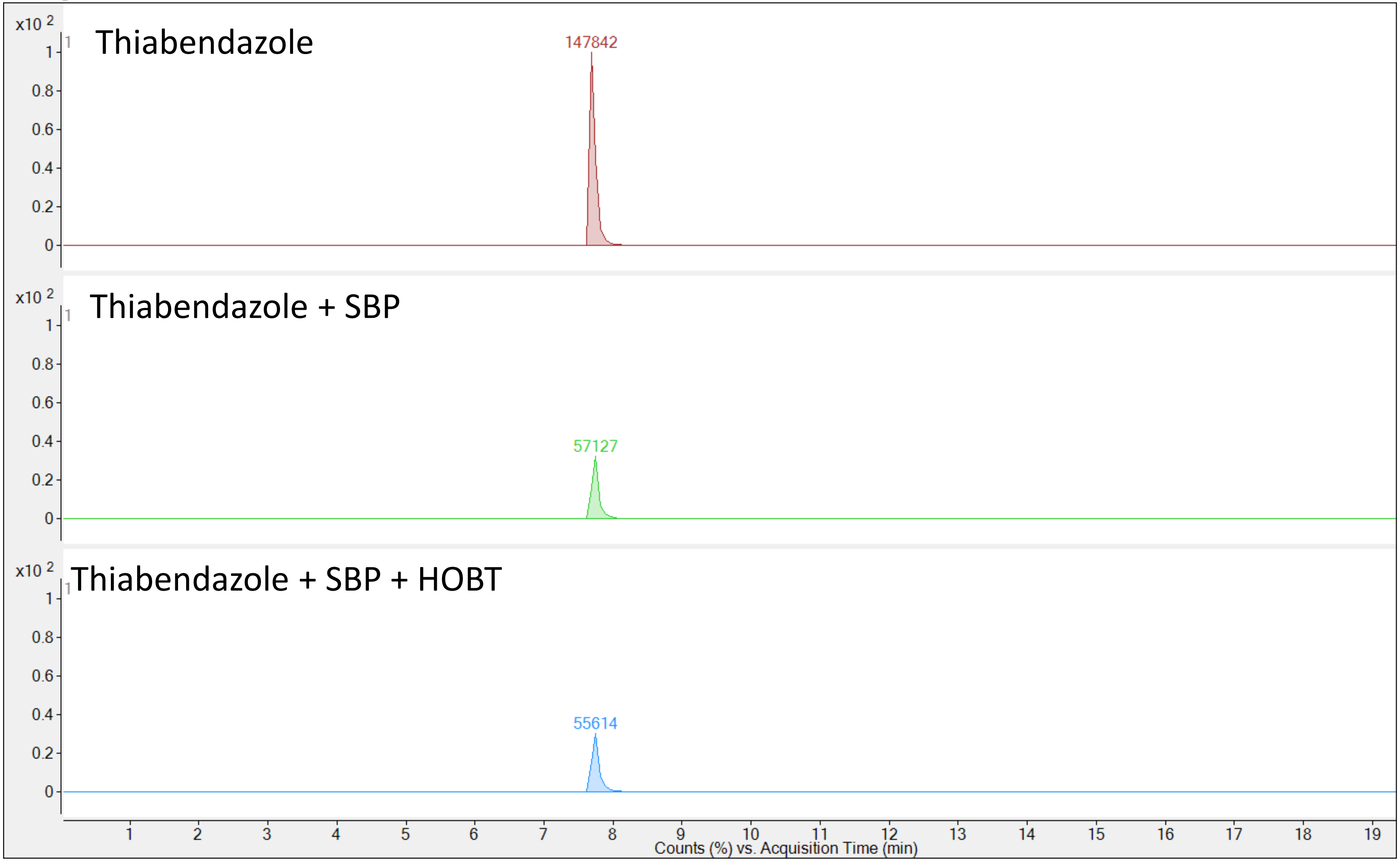

Figure 6C S: Effect of redox mediator on peroxidase-mediated pollutant degradation.
(C) Thiabendazole remaining after treatment with SBP enzyme with and without HOBT.
[Thiabendazole] = 2 ppm, [H_2O_2] = 0.1 mM added 3 times of 10 min interval, [HOBT] = 0.1 mM, pH = 4 with SBP enzyme and pH = 5 with MnP enzyme, [enzyme] = 0.36µM.

Fig 6D S

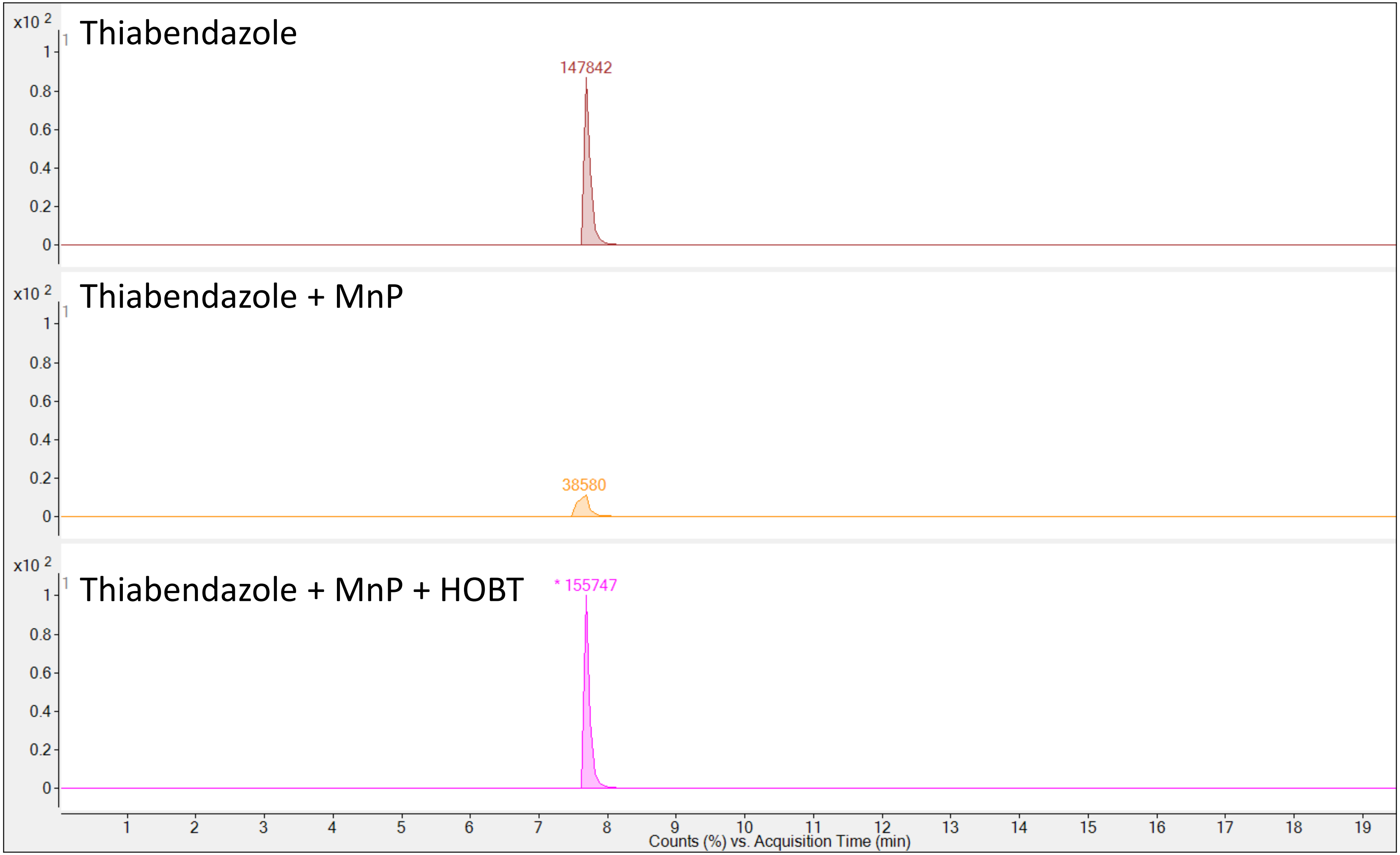

Figure 6D S: Effect of redox mediator on peroxidase-mediated pollutant degradation. .

(D) Thiabendazole remaining after treatment with MnP enzyme with and without HOBT.

[Thiabendazole] = 2 ppm, [H_2O_2] = 0.1 mM added 3 times of 10 min interval, [HOBT] = 0.1 mM, pH = 4 with SBP enzyme and pH = 5 with MnP enzyme, [enzyme] = 0.36µM.

Fig 7A S

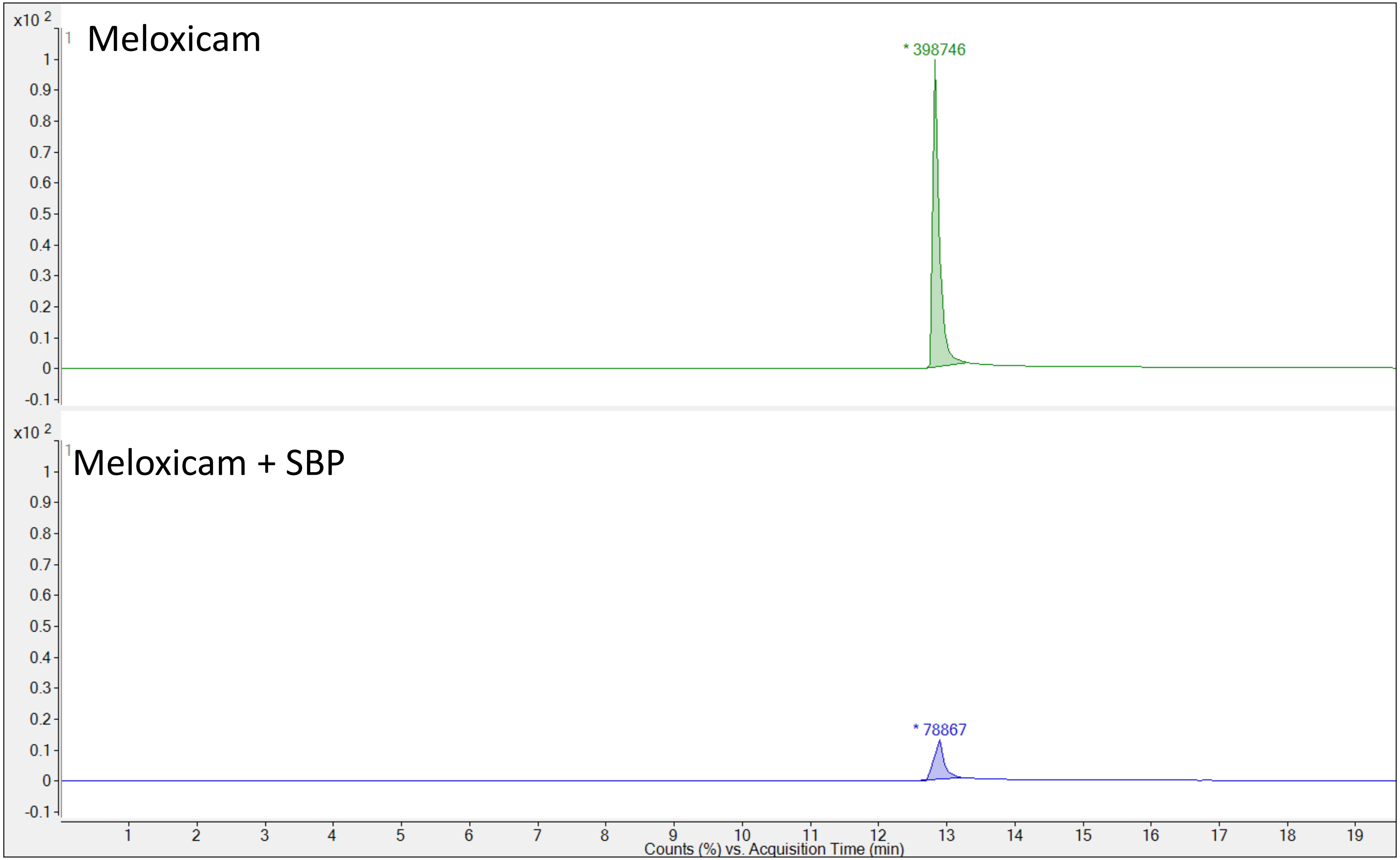

Figure 7A S: Degradation of pollutants spiked in real wastewater.
(A) Meloxicam remaining after treatment with SBP enzyme. [SBP] = 1.56µM, [H_2O_2] = 0.1 mM, [HOBT] = 50 µM, pH = 4.

Fig 7B S

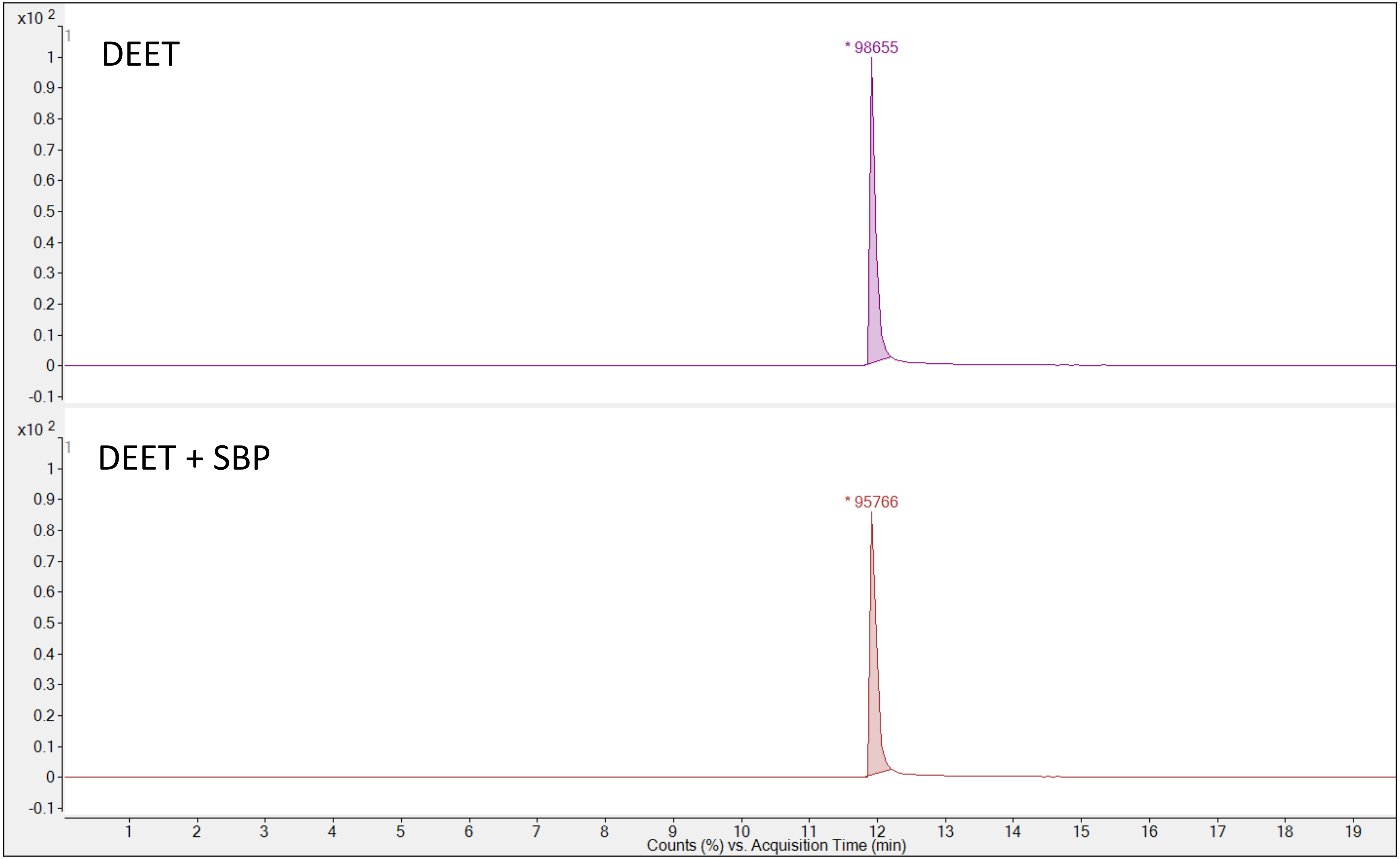

Figure 7B S: Degradation of pollutants spiked in real wastewater.
(B) DEET remaining after treatment with SBP enzyme. [SBP] = 1.56µM, [H_2O_2] = 0.1 mM, [HOBT] = 50 µM, pH = 4.

Fig 9B S

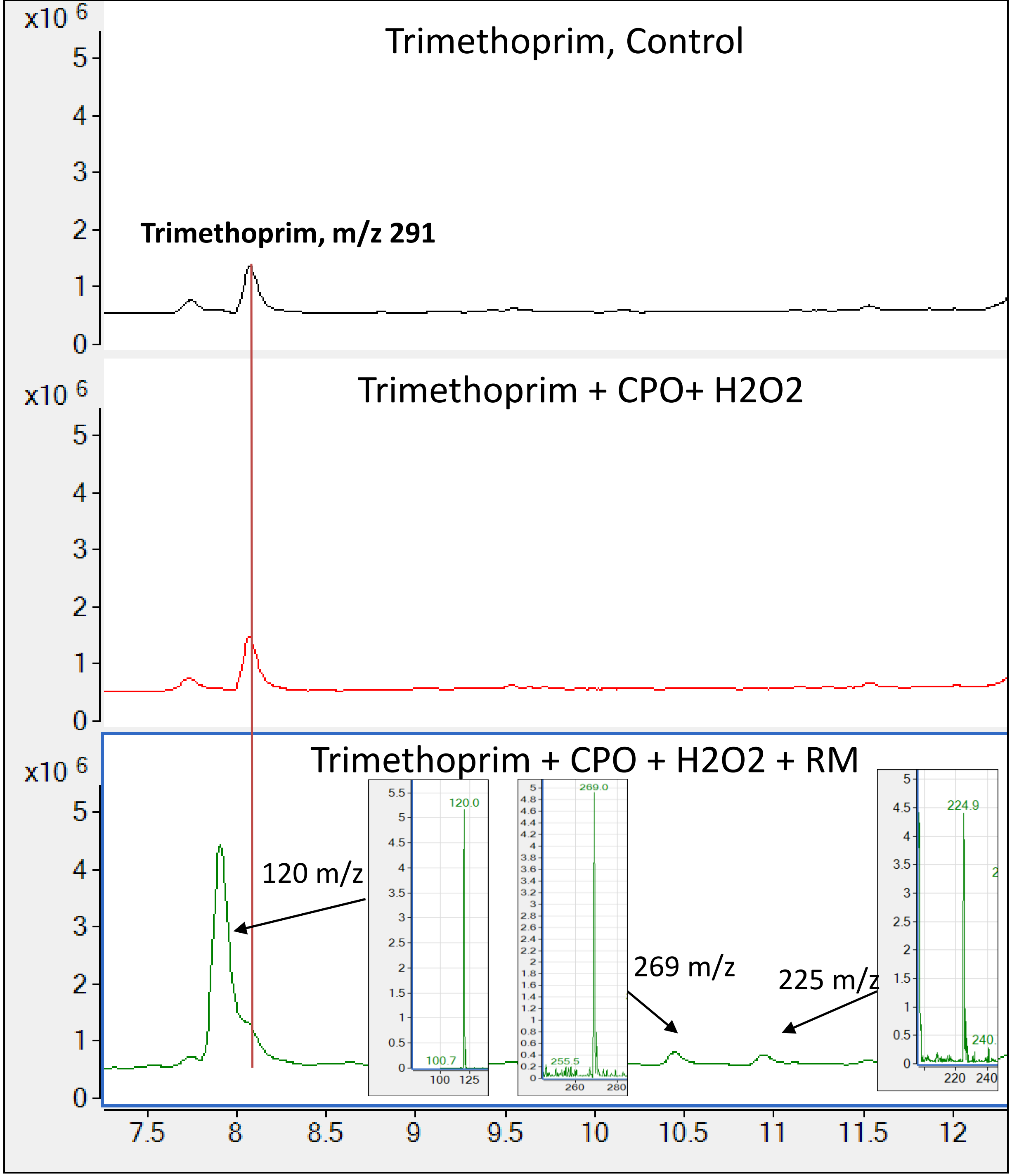

Figure 9B S: (B) Degradation of Trimethoprim by CPO + H_2O_2 + HOBT
